# Magnetic resonance imaging on brain structure and function changes in diabetic peripheral neuropathy

**DOI:** 10.3389/fneur.2023.1285312

**Published:** 2023-11-20

**Authors:** Li-qin Wang, Jin-huan Yue, Sheng-lan Gao, Dan-na Cao, Ang Li, Cai-liang Peng, Xiao Liu, Sheng-wang Han, Xiao-ling Li, Qin-hong Zhang

**Affiliations:** ^1^Department of Nursing Care, First Affiliated Hospital of Heilongjiang University of Chinese Medicine, Harbin, China; ^2^Shenzhen Frontiers in Chinese Medicine Research Co., Ltd., Shenzhen, China; ^3^Department of Acupuncture and Moxibustion, Vitality University, Hayward, CA, United States; ^4^Graduate School of Heilongjiang University of Chinese Medicine, Harbin, China; ^5^Division of CT and MRI, First Affiliated Hospital of Heilongjiang University of Chinese Medicine, Harbin, China; ^6^Servier (Beijing) Pharmaceutical Research & Development Co. Ltd., Beijing, China; ^7^Third Ward of Cardiology Department, First Affiliated Hospital of Heilongjiang University of Chinese Medicine, Harbin, China; ^8^Department of Pediatrics, First Affiliated Hospital of Heilongjiang University of Chinese Medicine, Harbin, China; ^9^Third Ward of Rehabilitation Department, Second Affiliated Hospital of Heilongjiang University of Chinese Medicine, Harbin, China; ^10^Heilongjiang University of Chinese Medicine, Harbin, China

**Keywords:** diabetes mellitus, diabetic peripheral neuropathy, voxel-based morphological analysis, functional magnetic resonance imaging, magnetic resonance spectroscopy

## Abstract

With the significant increase in the global prevalence of diabetes mellitus (DM), the occurrence of diabetic peripheral neuropathy (DPN) has become increasingly common complication associated with DM. It is particularly in the peripheral nerves of the hands, legs, and feet. DPN can lead to various adverse consequences that greatly affect the quality of life for individuals with DM. Despite the profound impact of DPN, the specific mechanisms underlying its development and progression are still not well understood. Advancements in magnetic resonance imaging (MRI) technology have provided valuable tools for investigating the central mechanisms involved in DPN. Structural and functional MRI techniques have emerged as important methods for studying the brain structures and functions associated with DPN. Voxel-based morphometry allows researchers to assess changes in the volume and density of different brain regions, providing insights into potential structural alterations related to DPN. Functional MRI investigates brain activity patterns, helping elucidate the neural networks engaged during sensory processing and pain perception in DPN patients. Lastly, magnetic resonance spectroscopy provides information about the neurochemical composition of specific brain regions, shedding light on potential metabolic changes associated with DPN. By synthesizing available literature employing these MRI techniques, this study aims to enhance our understanding of the neural mechanisms underlying DPN and contribute to the improvement of clinical diagnosis.

## Introduction

According to the International Diabetes Federation, the diabetes mellitus (DM) prevalence is expected to grow substantially in the coming years worldwide (1). The projection indicates that by 2030, the number of people affected by DM will reach a staggering 643 million individuals (1). This sharp increase in prevalence is alarming as it implies a significant rise in the number of people struggling with this chronic condition on a global scale. Furthermore, the projections suggest that by 2045, the number of individuals living with DM is forecasted to further escalate to an astonishing 783 million (1). Diabetic peripheral neuropathy (DPN) is the most common complication of DM, affecting 13 to 58% of diabetes patients (2, 3). It is characterized by symmetric, length-dependent sensory and motor neuropathy, resulting from metabolic and microvascular changes caused by factors like high blood glucose levels (4). This condition often manifests in peripheral symptoms such as numbness, tingling, pain, and abnormal sensations (5). DPN patients often exhibit poor motor performance, which increases the risk of falls, fractures, and severe disabilities (6). While DPN is commonly regarded as a disorder that affects the peripheral nervous system, recent evidence suggests that alterations in the central nervous system may also play a role in the progression of this disease (7, 8).

In recent years, advancements in imaging technology, including voxel-based morphometry (VBM), resting-state functional MRI (rs-fMRI), task-state functional MRI (ts-fMRI), and magnetic resonance spectroscopy (MRS), have enabled researchers to gain insights into the structural and functional alterations that take place in the brains of individuals suffering from DPN. These imaging techniques have significantly advanced our knowledge of DPN by uncovering intricate structural and functional changes in the brain. These insights have not only enhanced our understanding of the neural basis of DPN but have also provided crucial diagnostic tools for this condition. By further exploring and refining the applications of MRI in DPN research, researchers have made significant strides in understanding DPN from both a structural and functional perspective. These advances have deepened our knowledge about the impact of DPN on the brain, with the ultimate goal of shedding light on the underlying mechanisms of the disease.

## DNP study of brain structure

### VBM

**Figure 1:**
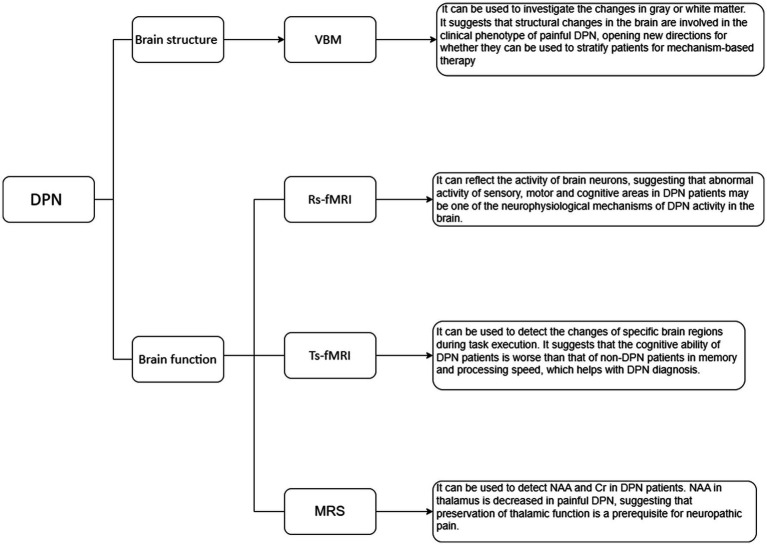
MRI Studies of brain structure and function in DPN.

VBM is a method that involves comparing the probability of grey matter or white matter between different groups by comparing voxels, sometimes described as the density or concentration of grey or white matter (9) (Figure 1). Its importance lies in its unbiased evaluation and comprehensive assessment of anatomical differences throughout the entire brain, rather than focusing on a specific structure (10, 11). In a study conducted by Selvarajah et al., 277 patients with type 1 DM (T1DM) and type 2 DM (T2DM), as well as 66 healthy volunteers, were included (12). The researchers conducted a study on participants with DPN and classified them into two phenotypes based on their pain response (12). One type consisted of participants with easily irritable (IR) nociceptors, while the other type comprised of subjects with non-irritable (NIR) nociceptors (12). The analysis of brain imaging data using FMRIB Software Library (FSL) revealed significant reductions in cortical thickness in the primary somatosensory and motor cortices of participants with both painful and painless DPN compared to healthy individuals and participants without DPN. The severity of DPN, as measured by neurophysiological assessments, was found to be correlated with the thickness of the motor cortex. Additionally, there was a decrease in the volume of the ventral basal ganglia in both painless and painful DPN. Further investigation focused on comparing the two nociceptor phenotypes within the painful DPN group. This study demonstrated significant changes in the structure of important brain regions responsible for sensing touch and pain in individuals with painless DPN and those with painful DPN (12). Specifically, the study observed alterations in the IR and NIR types of nociceptors, which are sensory receptors that respond to noxious stimuli. These findings suggest that modifications in the cerebral structure play a crucial role in determining the clinical manifestation of painful DPN (12). This exciting development opens up new avenues for research to explore whether these structural changes can be utilized to classify patients based on the underlying mechanisms of their pain. Ultimately, this could lead to the development of more targeted treatments tailored to each individual’s specific needs (12). In a separate study conducted by Hansen et al., grey matter volume (GMV) and cortical thickness were assessed using voxel-based/surface-based morphometry (13). The study involved adults with diabetic sensorimotor polyneuropathy (DSPN) and a control group. It was found that the total GMV was reduced in patients with DSPN compared to the control group (13). Furthermore, participants with painful neuropathy showed even more significant GMV loss compared to the control group. The researchers also explored the association between clinical features of DSPN and GMV loss. They found that features such as the severity of neuropathy and decreased concentration of N-acetyl aspartate/creatine (NAA/Cr) metabolite in the frontal lobes were associated with GMV loss in this cohort. Regional GMV loss was primarily observed in the bilateral thalamus, caudate, putamen, occipital lobes, and central precentral regions (13). In addition, cortical thickness reductions were observed specifically in frontal lobe regions (13). Overall, these studies provide evidence for significant structural alterations in the brain associated with diabetic neuropathy. Given the impact of clinical features on the observed total GMV loss, brain imaging can serve as a supplementary characterization tool for diabetic neuropathy.

In summary, GMV and the thickness of the cortex are two different measurements. The GMV reflects a combination of measurements including the volume, density, surface area of the cortex, sulcus, and gyrus patterns, as well as the thickness of the cortex. On the other hand, the thickness of the cortex only reflects the distance between the inner surface and outer surface of the gray matter on the cerebral cortex. The study conducted by Selverajah et al. found that participants with both painful and painless DPN had significantly reduced thickness in both the primary somatosensory and motor cortices (12). Additionally, Hansen et al.’s research compared the total GMV between the experimental and control groups (13), and these two studies complement each other in providing a comprehensive description of morphological changes in brain structures.

## DNP study of brain function

### Rs-fMRI

Rs-fMRI can provide valuable insights into the overall activity and has made breakthroughs in exploring brain activity and functional connectivity (14–17) (Figure 1). In rs-fMRI studies of DPN, the commonly used analysis methods are the amplitude of low frequency fluctuation (ALFF), fractional amplitude of low frequency fluctuation (fALFF), and functional connectivity (FC). Xin et al. (18) used rs-fMRI to study patients with DPN, non-DPN (NDPN), and normal subjects. We calculated the ALFF, fALFF, and the regional homogeneity (ReHo). Compared to the NDPN group, the DPN group demonstrated substantial reduction in ALFF variables in the right superior orbital gyrus (ORBsup), and medial superior frontal gyrus (SFGmed), and increment in ALFF variables in the left inferior temporal gyrus (ITG). The fALFF in the right SFGmed was also decreased in the DPN group. Receiver operating characteristic curve analysis showed substantial differences in ALFF/fALFF variables in the right SFGmed and average ALFF variables in the left ITG and right ORBsup between the DPN and NDPN subjects. Patients with DPN showed atypical patterns of brain activity in the sensory-motor and cognitive regions. These irregularities in brain activation could potentially serve as critical indicators of underlying neurophysiological mechanisms responsible for DPN. Cauda et al. (19) studied resting-state FC (rsFC) in DNP patients and compared them to a healthy control group. The connectivity of the dorsal and ventral attention networks and the connectivity of the dorsal anterior cingulate cortex (ACC) were generally decreased in DNP patients, which is related to enhanced processing. In the DNP group, the length of functional connections was generally shortened: the Euclidean distance between voxels connected in the DNP group was substantially shorter than that in the healthy control group across all examined networks. The top-down attention network involved in controlling attention was impaired in patients with diabetic neuropathy. Consistent with previous studies, chronic pain can disrupt the synchronization of common brain areas involved in self-monitoring, pain processing, and salience detection. Croosu et al. (20) conducted a study using rs-fMRI to investigate the brain activity and connectivity patterns in different groups of patients. The study included patients with T1DM and DPN, patients with T1DM and painless DPN, T1DM patients without DPN, and a group of healthy individuals as control subjects. Functional brain connectivity between the thalamus, posterior cingulate cortex, and insula was analyzed using seed-based connectivity analysis, and connectivity *z*-scores were correlated with peripheral nerve function measurements and pain scores. Overall, compared to DPN pain patients and healthy controls, T1DM patients without DPN showed increased connectivity between the thalamus and motor areas, as well as between the posterior cingulate cortex and motor areas. Poorer peripheral nerve function and higher pain scores were associated with lower connectivity between the thalamus and posterior cingulate cortex. Based on thalamic connectivity, the phenotype of T1DM can be divided into painful/painless DPN and T1DM without DPN. The current research findings support the use of functional MRI for phenotype analysis, which may help in early detection and prevention of neuropathic complications.

### Ts-fMRI

Ts-fMRI is a technique commonly used in neuroscience research to study the activation patterns of specific brain regions and circuits during different task execution processes (21–23) (Figure 1). In a study conducted by Ni et al. (24), the researchers examined a group of healthy individuals and participants with T2DM using a combination of neuroconduction tests, detailed cognitive assessments, olfactory behavioral tests, and odor-induced fMRI. The patients with T2DM were further assigned to NDPN and DPN groups. The researchers found that the DPN group demonstrated significant reductions in memory and processing speed scores, olfactory recognition and memory scores, when compared to the NDPN group. This suggests that individuals with DPN may experience cognitive deficits. Furthermore, the researchers discovered that the DPN group demonstrated decreased activation in the left frontal lobe, which is involved in various cognitive processes such as executive functions and decision-making. Additionally, there was a decreased seed-based FC in the right insula in DPN patients. The insula is a brain region implicated in emotional processing and interoception. These findings indicate that individuals with DPN have poorer cognitive abilities in terms of memory and processing speed compared to those with NDPN. Interestingly, the study also revealed that cognitive impairment can be predicted through olfactory behavioral tests and electrophysiological examinations, highlighting the potential utility of these non-invasive assessments in diagnosing and monitoring cognitive deficits in individuals with T2DM.

### MRS

1H-MRS is a non-invasive imaging technique that allows researchers to study the neurochemistry of specific areas in the brain known as voxels (25, 26). By using different acquisition methods, researchers can detect various low molecular weight metabolites, such as NAA and Cr (27) (Figure 1). Sloan et al. (28). utilized 31P MRS to explore the bioenergetics in the primary somatosensory (S1) cortex of a group of patients with painful and painless DPN. They measured the ratio of phosphocreatine (PCr)/adenosine triphosphate (ATP), which serves as a measure of energy expenditure. The results exerted a substantial reduction in the PCr/ATP ratio in the group with painless DPN compared to the painful DPN group. This suggests that painful DPN patients have a higher energy consumption in the S1 cortex. Additionally, the relationship between PCr/ATP and neuropathic pain measurements indicates that S1 bioenergetics is associated with the severity of neuropathic pain. The findings suggest that S1 cortex bioenergetics could serve as a biomarker for painful DPN and may have the potential to be a target for therapeutic interventions. Another study by Sorensen et al. (29). used 1H-MRS to examine the brains of participants with and without painful DPN. The left thalamus, ACC, and dorsolateral prefrontal cortex (DLPFC) were investigated by 1H-MRS. The diabetic patients exhibited reduced levels of NAA and Cr in the DLPFC compared to the control group. In the thalamus, the group with painful DPN showed lower NAA levels compared to the group without pain. These findings signify that NAA and Cr are different in the brains of DM patients compared to the control subjects, and the decrease in thalamic NAA levels in individuals with painful neuropathy could potentially contribute to the development of pain in certain cases. Overall, 1H-MRS is a valuable technique for studying the neurochemistry of specific brain regions and has provided insights into the differences in neuronal functionality and metabolite levels between individuals with and without painful DPN. These findings help to elucidate the intricate mechanisms of neuropathic pain and may inform the development of targeted interventions for pain management in individuals with DPN. Hansen et al. (30) conducted a cross-sectional study on patients with DSPN in T1DM and healthy controls. They utilized MRS to evaluate the levels of NAA/Cr in the thalamus. The results showed that compared to healthy individuals, there was a positive correlation between estimated thalamic volume and NAA/Cr levels in the thalamus. In patients with T1DM and severe DSPN, there was thalamic atrophy, which was associated with a decrease in NAA/Cr levels. This suggests that there is both structural loss and functional impairment in the thalamus, which may ultimately contribute to a deeper understanding of the pathophysiological processes that underlie the development and progression of DSPN.

## Limitations

Currently, there are still several limitations in MRI studies on DPN. Firstly, the majority of studies conducted so far have been limited to a single healthcare center, which might restrict the generalizability of their findings. Moreover, the limited size of the samples used in these studies might potentially impact the accuracy and reliability of the findings. Secondly, another limitation lies in the relatively simplistic approach used for patient grouping in these MRI studies. This means that the researchers have mainly categorized patients into broad groups based on general DPN symptoms without considering the heterogeneity of the disease and its various stages. Consequently, this oversimplified patient grouping might overlook critical differences between individuals and impede the identification of specific imaging patterns associated with different stages or types of DPN. Furthermore, it should be noted that only one MRI technique has been commonly employed for experimental analysis in these studies. This single technique might fail to capture the comprehensive neuropathic changes occurring in DPN patients. It overlooks the rich diversity of mechanisms and manifestations in DPN pathology, which could be better elucidated with a combination of multiple MRI modalities such as fMRI, or MRS. By combining the information generated by these different MRI techniques, researchers can obtain a multi-dimensional perspective of DPN, which can significantly enhancing our knowledge of the disease mechanisms involved. In summary, the current limitations in MRI studies on DPN include the restriction of single-center and small-scale studies, simplified patient grouping strategies, and the limited utilization of multiple MRI techniques. Overcoming these limitations through larger multi-center studies, more sophisticated patient stratification, and the integration of diverse MRI methods would undoubtedly offer a more detailed and holistic understanding of DPN and facilitate the development of more effective diagnostic and therapeutic strategies.

## Summary

In summary, MRI technology can clearly reflect the alterations in brain structure and function in DPN patients. In DPN brain structure studies, significant structural changes in specific key body movement or nociceptive brain areas of painless and painful DPN have been discovered using VBM, which can be used as a supplementary representation of diabetic neuropathy. In patients with DPN, there is evidence of deviation from normal brain activity in specific regions related to sensory motor functions and cognitive processes. This abnormal brain activity may be considered as one of the underlying neurophysiological mechanisms contributing to the manifestation of DPN. Reduced NAA in the thalamus of painful DPN may explain the origin of pain in some cases.

In the future, it is necessary to further study the changes in DPN brain tissue by combining multiple methods, multi-center, and longitudinal MRI analysis. Additionally, the application and development of techniques such as neural networks, machine learning, and deep learning in the field of image processing can improve the accuracy of DPN diagnosis.

## Author contributions

L-qW: Conceptualization, Data curation, Funding acquisition, Resources, Validation, Visualization, Writing – original draft, Writing – review & editing. J-hY: Conceptualization, Resources, Visualization, Writing – original draft, Writing – review & editing. S-lG: Data curation, Resources, Validation, Visualization, Writing – review & editing. D-nC: Funding acquisition, Resources, Validation, Visualization, Writing – review & editing. AL: Methodology, Resources, Validation, Visualization, Writing – original draft, Writing – review & editing. C-lP: Resources, Validation, Visualization, Writing – review & editing. XL: Resources, Validation, Visualization, Writing – review & editing. S-wH: Funding acquisition, Resources, Validation, Visualization, Writing – review & editing. X-lL: Conceptualization, Funding acquisition, Investigation, Project administration, Supervision, Validation, Visualization, Writing – original draft, Writing – review & editing. Q-hZ: Conceptualization, Data curation, Investigation, Project administration, Supervision, Validation, Visualization, Writing – original draft, Writing – review & editing.
